# Determination of Antiviral Drugs and Their Metabolites Using Micro-Solid Phase Extraction and UHPLC-MS/MS in Reversed-Phase and Hydrophilic Interaction Chromatography Modes

**DOI:** 10.3390/molecules26082123

**Published:** 2021-04-07

**Authors:** Luboš Fical, Maria Khalikova, Hana Kočová Vlčková, Ivona Lhotská, Zuzana Hadysová, Ivan Vokřál, Lukáš Červený, František Švec, Lucie Nováková

**Affiliations:** 1 Department of Analytical Chemistry, Faculty of Pharmacy in Hradec Králové, Charles University, Akademika Heyrovského 1203, 500 05 Hradec Králové, Czech Republic; ficall@faf.cuni.cz (L.F.); khalikom@faf.cuni.cz (M.K.); VLCKH3AA@faf.cuni.cz (H.K.V.); lhotski@faf.cuni.cz (I.L.); hadysovz@faf.cuni.cz (Z.H.); svecfr@faf.cuni.cz (F.Š.); 2 Department of Pharmacology and Toxicology, Faculty of Pharmacy in Hradec Králové, Charles University, Akademika Heyrovského 1203, 500 05 Hradec Králové, Czech Republic; vokral@faf.cuni.cz (I.V.); cervenyl@faf.cuni.cz (L.Č.)

**Keywords:** UHPLC-MS/MS, hydrophilic interaction chromatography, reversed phase, antiviral drug, microextraction, solid phase extraction

## Abstract

Two new ultra-high performance liquid chromatography (UHPLC) methods for analyzing 21 selected antivirals and their metabolites were optimized, including sample preparation step, LC separation conditions, and tandem mass spectrometry detection. Micro-solid phase extraction in pipette tips was used to extract antivirals from the biological material of Hanks balanced salt medium of pH 7.4 and 6.5. These media were used in experiments to evaluate the membrane transport of antiviral drugs. Challenging diversity of physicochemical properties was overcome using combined sorbent composed of C_18_ and ion exchange moiety, which finally allowed to cover the whole range of tested antivirals. For separation, reversed-phase (RP) chromatography and hydrophilic interaction liquid chromatography (HILIC), were optimized using extensive screening of stationary and mobile phase combinations. Optimized RP-UHPLC separation was carried out using BEH Shield RP18 stationary phase and gradient elution with 25 mmol/L formic acid in acetonitrile and in water. HILIC separation was accomplished with a Cortecs HILIC column and gradient elution with 25 mmol/L ammonium formate pH 3 and acetonitrile. Tandem mass spectrometry (MS/MS) conditions were optimized in both chromatographic modes, but obtained results revealed only a little difference in parameters of capillary voltage and cone voltage. While RP-UHPLC-MS/MS exhibited superior separation selectivity, HILIC-UHPLC-MS/MS has shown substantially higher sensitivity of two orders of magnitude for many compounds. Method validation results indicated that HILIC mode was more suitable for multianalyte methods. Despite better separation selectivity achieved in RP-UHPLC-MS/MS, the matrix effects were noticed while using both chromatographic modes leading to signal enhancement in RP and signal suppression in HILIC.

## 1. Introduction

Antiviral drugs are an important class of compounds because many viruses can cause life-threatening diseases, as we are now witnessing with the COVID-19 pandemic. Antiviral drugs can act against viruses at different stages of the viral life cycle, including (i) inhibitors of virion fusion or entry, (ii) inhibitors of uncoating, (iii) inhibitors of integrase, (iv) inhibitors of nucleic acid synthesis, (v) protease inhibitors, and (vi) neuraminidase inhibitors [1]. Most antiviral drugs affect the viral synthesis step, thus, important groups involve the reverse transcriptase inhibitors and the protease inhibitors, acting in the viral replication and maturation step, respectively. A variety of structurally different antiviral drugs has been developed to target these different stages and also different types of viruses, including generally four major groups of (i) herpes viruses, (ii) respiratory viruses, (iii) hepatitis A, B, and C viruses (HAV, HBV, HCV), and (iv) human immunodeficiency virus (HIV) [1,2].

To 2020, more than 100 antiviral drugs have been approved by US Food and Drug Administration (FDA) [2,3]. Most recently approved antiviral drugs belong among anti-HIV and anti-hepatitis C (HCV) drugs in addition to drugs combating newly emerging viruses, such as Ebola virus and SARS-CoV-2 [3,4]. As the development of a new antiviral drug is very time-consuming and costly, the strategy of approvement of drugs previously registered in different indications for the new treatment purpose called drug repurposing, is currently also adopted in antiviral drug development [5,6]. Well-established doses and regimens are known for these drugs, their side effects and prevention are defined, synthetic routes for their preparation were implemented, and safety and quality assurance achieved. Therefore, approval of a new indication is usually faster, easier, and less expensive [6].

To support the evaluation of both, new antiviral drugs and repurposed antiviral drugs, their quantitative analysis in biological materials is of key importance to determine bioavailability, drug metabolism, and other pharmacokinetic parameters, as well as to allow therapeutic drug monitoring in later stages. Oral intake of antiviral drugs is the most common route of drug administration because it is considered the safest, most convenient, and highly economical. However, absorption of the drug from the gut is significantly affected by the presence of the intestinal barrier functioning as a selective filter for xenobiotics, including drugs. This barrier contains a range of tools, including xenobiotic-metabolizing enzymes and efflux transporters that prevent xenobiotics from entering the systemic circulation [7]. Concomitant oral administration of multiple drugs that are substrates, inducers, or inhibitors of these enzymes and/or transporters can subsequently result in drug-drug interactions (DDI) and hence in altered plasma levels [7]. DDI can occur in both directions meaning failure of the therapy in case of decreased plasma level of the drug below the therapeutic level and toxic effect if this level is exceeded. The risk of DDI is more common in aging patients characterized by comorbidities and polypharmacy [8].

Moreover, combination therapy using several antivirals from different classes with different mechanisms of action and therapy optimization are common in complex HIV treatment and in some cases of HCV treatment [9]. Anti-HIV and anti-HCV drugs are the typical examples of drugs administered orally where a high risk of DDI on intestinal efflux transporters can be expected [10]. Regulatory authorities as FDA and European Medicines Agency (EMA) are aware of this risk. Therefore, they recommend cell line-based assays (e.g., Caco-2, MDCK) to reveal the DDI in preclinical research [11,12]. New, more complex promising models as precision-cut intestinal slices (PCIS) are also emerging in this process [10]. As anti-HIV and anti-HCV drugs are administered in combination therapy, preclinical research methods must be complex enough to reveal the majority of possible DDI in different experimental models. To support the antiviral therapy aspects discussed above, multianalyte analytical methods allowing to monitor a large spectrum of antivirals in a single analytical run with high sensitivity and selectivity are beneficial. Although it is unlikely that numerous antivirals can be simultaneously present in biological sample since typical combinations involve two to three antivirals, a unique multianalyte method that can separate them all in a single run is desirable. It would allow separation of any combination without carrying out often tedious optimization of a dedicated methods enabling switching among these combinations and testing different compounds for DDI. Therefore, multianalyte methods are more suitable than single or several-analyte methods.

High-performance liquid chromatography coupled to tandem mass spectrometry (HPLC-MS/MS) is the method of choice that meets all these requirements [1]. The bioanalytical methods used in the analysis of antiviral drugs were recently summarized in two comprehensive review papers we published that focused on individual groups of antivirals [1,9] and covered the period of 2000–2017. In addition, the most recent review article described the antivirals against COVID-19 [13]. The importance of multianalyte bioanalytical methods [14,15,16,17,18,19,20] has been emphasized already in our above stated review papers. Various newly published research papers have also demonstrated successful use of LC-MS/MS methods for the simultaneous analysis of 7–16 antiviral agents [21,22,23,24,25,26]. Moreover, detailed MS/MS characterization of antivirals addressing selected reaction monitoring (SRM) data and also structure elucidation of individual fragments have been presented in extensive review articles for antivirals for HIV treatment [27], antivirals against hepatitis [28], and against herpes, influenza, and other viral infections [29]. Despite quite an important number of reported methods, simultaneous analysis using single analytical approach for sample preparation and separation of current antivirals remains challenging due to the structural diversity of antiviral drugs as shown in Table 1. For example, their log P range of −3.44 to 6.71 covers compounds from highly hydrophilic, such as tenofovir and its metabolites, zidovudine, and didanosine, to highly lipophilic, such as ledipasvir, velpatasvir, ritonavir, and rilpivirine. Acid-base properties include all types of compounds, i.e., neutral, acidic, and basic. While the separation step is facilitated by the availability of a wide spectrum of current stationary phases and gradient elution, the sample preparation step addressing such structural variability remains tricky. As a result, fast and efficient but non-selective protein precipitation sample preparation step was adopted in most of the published multianalyte works.

Our current study aimed to develop a complete multianalyte analytical approach for determining 21 selected antiviral drugs and their metabolites in the biological medium used to evaluate DDI on membrane efflux transporter P-glycoprotein using the Caco-2 cell line and PCIS-based methods. 

We compared two separation approaches: (i) reversed-phase HPLC and (ii) hydrophilic interaction chromatography in terms of retention, separation selectivity, and sensitivity in MS/MS detection. It is worth noting that HILIC has not been used in multianalyte methods for antivirals yet. We used micro-solid phase extraction in pipette tips (µ-SPE-PT) as a more selective alternative to the protein precipitation reported most often previously. Optimization of composition of the extraction sorbent and individual µ-SPE-PT steps allowed to design tailor-made tool for an effective simultaneous extraction even for a group of target analytes featuring an important structural diversity. This unique feature of our miniaturized SPE approach is difficult to achieve using more conventional SPE approaches, because the commercially available sorbents have limited flexibility. The UHPLC-MS/MS methods including µ-SPE-PT sample preparation were validated for both HILIC and RP with two biological media. The challenges, benefits, and drawbacks of both approaches were also defined.

## 2. Results and Discussion

RP-UHPLC-MS/MS has been a method of choice in most previously published methods for the analysis of antiviral drugs [1,9]. However, HILIC is known to provide orthogonal separation selectivity and enhanced sensitivity when coupled with ESI-MS/MS [30]. Moreover, it can be helpful in addressing several issues, such as separating interferences and/or the matrix effects. Therefore, our study optimized both methods in detail using extensive screening of stationary and mobile phases in both modes.

### 2.1. UHPLC-MS/MS Method Development in Reversed Phase Mode

Optimization of ion source conditions and SRM transitions for individual antiviral drugs was carried out in the first step of method development. Full scan MS spectra and product ion spectra were measured in both ESI^+^ and ESI^−^ for all target compounds. Indeed, most of the compounds exhibited ionization in both tested modes under RP-UHPLC-MS/MS conditions except for abacavir, atazanavir, efavirenz, and tenofovir disoproxil that did not ionize in ESI^−^. Ledipasvir and velpatasvir exhibited higher intensity for doubly charged ions [M + 2H]^2+^ in ESI^+^ compared to their protonated counterparts [M + H]^+^. This phenomenon was not observed in ESI^−^. Besides this exception, precursor ions for SRM were always protonated [M + H]^+^ and deprotonated [M − H]^−^ molecules. Three fragments with the highest abundance were selected for each precursor in the fragmentation spectra. These were used to set individual SRM transitions and optimize collision energies and cone voltages for each compound. All other ion source parameters were also determined for the whole set of antiviral drugs, but the resulting settings shown in Section 3.2 were compromise values providing an overall high MS response for all analytes. Finally, the sensitivity of the most intense SRM transitions was compared in ESI^+^ and ESI^−^ using a set of calibration solutions and evaluation of the lowest detectable concentration. The resulting SRM transitions are shown in Table 1 and were used in all optimization steps. Although these experiments were carried out independently before the publication of the series of recent articles focused on the fragmentation of antiviral drugs, our results are in very good agreement with them. Most of the fragments used in our SRM transition method correspond well with the findings of Niessen [27,28] with only some exceptions. The fragment of *m*/*z* 176.1 was found and tuned in our method development for tenofovir alafenamide and tenofovir disoproxil. However, a higher sensitivity was achieved with another fragment of *m*/*z* 270.0 that is also another fragment typical of tenofovir structure [27]. Different fragments were used as well in SRM transitions of velpatasvir and ledipasvir, but again, these corresponded well to expected fragmentation patterns [28]. Higher sensitivity was achieved in ESI^−^ for rilpivirine being a finding that has not been reported yet. Similarly, the MS/MS data for glecaprevir has not been reported neither [28].

The generic gradient elution method shown in Section 3.2 was used to screen six RP stationary phases, including BEH Shield RP18, BEH Phenyl, BEH C18, CSH Fluoro-phenyl, CSH Phenyl-hexyl, and CSH C18 using 25 mmol/L formic acid in the aqueous component. Several critical separations were defined in the target set of compounds, including tenofovir and all its derivatives, because the *m*/*z* typical of tenofovir molecule was observed in all these spectra, but also abacavir (*m*/*z* 286.1542) and tenofovir (*m*/*z* 287.0783) and its derivatives due to the possibility of the interference with the abacavir M + 1 isotope. The separation was also required for tenofovir disoproxil (*m*/*z* 519.173) and boceprevir (*m*/*z* 519.3421) provided that triple quadrupole mass analyzer enabled only unit mass resolution. Thus, such close masses cannot be discriminated. While all tested stationary phases enabled elution of all tested antiviral drugs, some of the critical pairs were not separated using CSH fluorophenyl (tenofovir/tenofovir monoester/tenofovir disoproxil), CSH Phenyl (tenofovir/abacavir), BEH phenyl (tenofovir monoester/abacavir), and CSH C18 (abacavir/tenofovir) stationary phases. Thus, phenyl moiety and CSH sorbents were found to be less useful for the separation of target antiviral drugs and for this kind of multianalyte methods. Stationary phases with C_18_ functionality attached to bridged ethyl hybrid support produced better separation selectivity for these critical pairs and also resulted in an overall lower number of coelutions. This is beneficial in the case of ESI-MS/MS detection, where coelutions may result in matrix effects. Both BEH C18 and BEH Shield RP18 exhibited very good separation selectivity and allowed obtaining symmetrical peak shapes with slightly better results for the latter, which was selected for further experiments.

In the next step, the most common LC-MS additives including formic acid and acetic acid were tested besides ammonia and buffers. These experiments enabled evaluation of effects of both acidic and basic pH using ammonium formate pH 3 and ammonium acetate pH 9. All these additives were tested at four different concentrations levels of 1, 5, 10, and 25 mmol/L with the goal to evaluate especially mass spectrometry response, but also separation selectivity and peak shapes. Formic acid, acetic acid, and ammonia led to the best results at 25 mmol/L concentration level, while ammonium buffers provided better results at 5 mmol/L concentration. Although the higher response was monitored with ammonia and ammonium acetate at pH 9 for some analytes, such as for example tenofovir and its derivatives, zidovudine, maraviroc, ledipasvir protonated molecule, atazanavir, and boceprevir, we observed a complete loss of response for didanosine and efavirenz and an important decrease in response for daclatasvir. Rilpivirine showed a decrease in response only with ammonia. Moreover, basic pH conditions resulted in reduced separation selectivity. The basic conditions resulted in many coelutions and only 7 completely separated peaks using 5 mmol/L ammonium acetate pH 9 and 8 peaks in 25 mmol/L ammonia. Slightly better results were observed at acidic pH with 13 completely separated peaks for 5 mmol/L ammonium formate pH 3, 11 peaks for 25 mmol/L acetic acid, and 13 completely resolved compounds out of 21 tested target compounds for 25 mmol/L formic acid. Therefore, 25 mmol/L formic acid with acetonitrile was used in gradient elution with no need to modify separation conditions further keeping the aim to use effectively the separation space.

**Figure 1:**
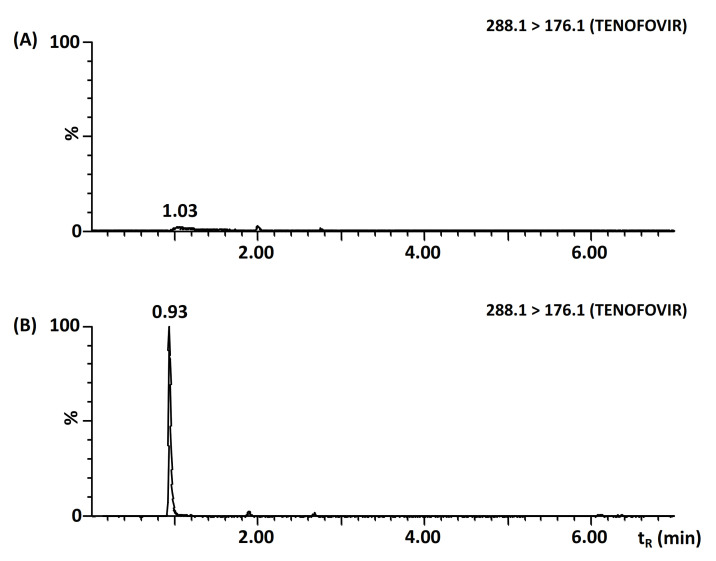
Comparison of RP-UHPLC-MS/MS chromatograms of tenofovir without (**A**) and with (**B**) the addition of 0.1% medronic acid in both components of the mobile phase. Chromatographic conditions were described in Section 3.2.

However, using these and all tested RP conditions, the tenofovir peak shape changed unexpectedly and irregularly. We observed strong tailing and substantial loss of sensitivity, which hindered achieving the desired method sensitivity and repeatable quantitative results. Due to the presence of a phosphate group in the tenofovir structure, we suspected its interaction with trace amounts of metals in the chromatographic system that can also vary randomly. Medronic acid has been reported to mitigate this problem due to its binding capability of these metal ions [31]. The addition of medronic acid was tested in two ways: (i) in the injected sample and (ii) in both components of the mobile phase. However, the simple injection did not allow the complete removal of the trace metals and resulted in tenofovir peak tailing and irreproducible elution. Thus, the addition of 0.1% medronic acid in both mobile phase components and careful system saturation was necessary to ensure symmetric peak shape and repeatable elution for tenofovir as shown in Figure 1. Apparently, the effect of the addition of medronic acid in the mobile phase on the separation selectivity, peak shapes, and MS response needed to be examined carefully. Due to the acidic nature of medronic acid and the original water component of the mobile phase, the effect was negligible in terms of retention time (RSD < 0.4 %) and peak shape for all target analytes, besides the problematic tenofovir. Moreover, the effect of medronic acid on peak area was negligible or positive in most cases, resulting in no change or in an important increase in response for some analytes, such as velpatasvir, ledipasvir, and saquinavir. Only five analytes including didanosine, zidovudine, daclatasvir, maraviroc, and rilpivirine experienced about 50% decrease in signal that had to be tolerated at the cost of the method universality. However, if tenofovir is not the subject of the study, the original conditions without addition of medronic acid can be employed as well.

**Figure 2:**
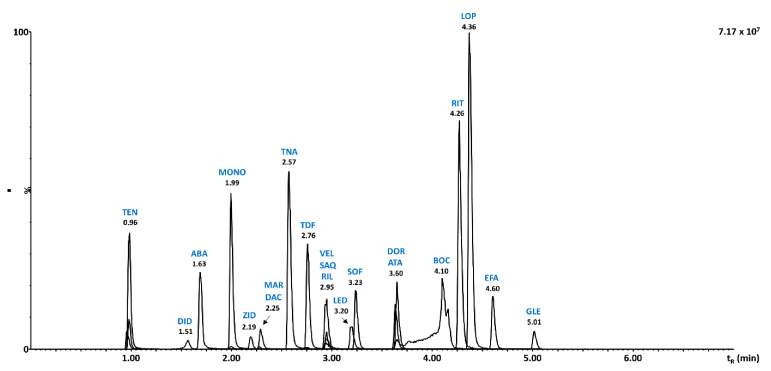
RP-UHPLC-MS/MS chromatogram of 21 antiviral drugs using optimized separation conditions: BEH Shield RP18 stationary phase, gradient elution using 25 mmol/L formic acid in water with the addition of 0.1% of medronic acid and ACN with addition 0.1% medronic acid, gradient program from 5% ACN to 98% ACN in 5 min, injection volume 2 µL, and flow rate 0.3 mL/min.

Important differences in the physicochemical properties and thus in solubility of target antiviral drugs did not allow using a water solution for the injection in the chromatographic system. Increasing percentage of ACN in dissolution solvent from 5 to 50% resulted in an enhanced MS response. However, the presence of ACN resulted in distorted peaks of early eluted analytes of saquinavir, tenofovir alafenamide, and zidovudine. As a result, 20% ACN was selected as a compromise for peak shapes and signal intensity. The final separation of 21 antiviral drugs under all optimized conditions described in Section 3.2 is shown in Figure 2. A compromised peak shape was obtained for boceprevir. This problem has been observed when all six tested stationary phases were used and also in the case of mobile phase additives except for ammonia. Unfortunately, compromises are always necessary for multiplex multianalyte methods focused on analytes of very different physicochemical properties and can be accepted if the method provides reliable quantitative results.

### 2.2. UHPLC-MS/MS Method Development in HILIC Mode

The MS tuning experiments and SRM optimization in both ESI^+^ and ESI^−^ were repeated in HILIC mode, with notable differences obtained only in capillary voltage (see Section 3.2) and cone voltages. Thus, the same SRM transitions using the same polarities with different cone voltage values for some analytes shown in Table 1 were used in HILIC. The preliminary evaluation of sensitivity using standard solutions in HILIC mode confirmed substantially higher sensitivity of several orders of magnitude for some compounds compared to RP.

Ten HILIC stationary phases were screened with four different mobile phases due to the more complex HILIC separation mechanism and a lower predictability of its behavior. Higher concentrations of 10 and 25 mmol/L additives were used since a lower percentage of the aqueous component is typical of the HILIC gradient program. Overall evaluation including all stationary phases indicated that buffers always enabled better chromatographic performance compared to formic and acidic acid. Indeed, tailing peaks and reduced separation selectivity were observed when using only acid, either formic or acetic, as the aqueous component. Generally, 25 mmol/L buffers provided better results in both separation and MS response than 10 mmol/L counterparts. The best score in terms of the number of separated peaks was obtained using core-shell-based silica stationary phases Cortecs HILIC and Ascentis Express HILIC, and hybrid silica BEH HILIC. Overall, limited separation selectivity was observed compared to RP separations, but the defined critical separations (see Section 2.1) were accomplished using the three selected stationary phases.

Overall, elution of all target antiviral drugs was achieved also in HILIC with the exception of tenofovir. The elution of the tenofovir peak was even more critical in HILIC than in RP. Indeed, without the addition of medronic acid, the peak of tenofovir was not eluted from most of the tested stationary phases. Its peak was extremely tailing in the cases where it was eluted. However, the addition of medronic acid in both mobile phase components allowed efficient elution and separation of tenofovir even in HILIC.

**Figure 3:**
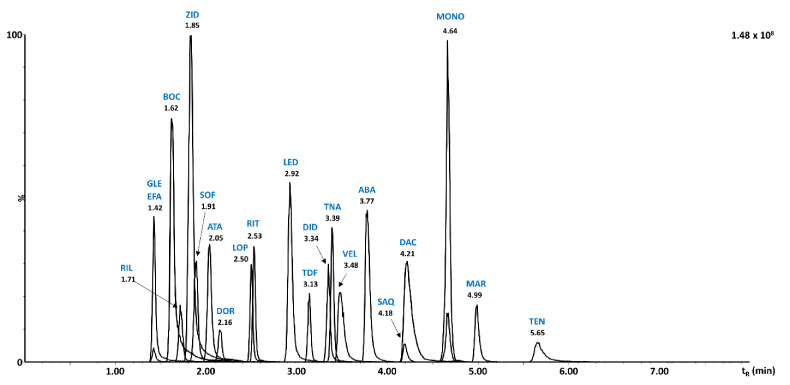
HILIC-UHPLC-MS/MS chromatogram of 21 antiviral drugs using optimized separation conditions: Cortecs HILIC column, 25 mmol/L ammonium formate pH 3 (mobile phase A) and ACN (mobile phase B) in gradient elution with the gradient program 98% to 50% B in 5 min. Medronic acid (0.1 %) was added to both mobile phase components. Injection volume 2 µL and flow rate 0.3 mL/min.

The effect of dilution solvent was also substantially more critical in HILIC compared to RP. Some of the early eluting peaks of doravirine, lopinavir, sofosbuvir, ritonavir, and atazanavir were affected most significantly. Indeed, already the use of 95% ACN substantially compromised the peak shapes of these analytes and resulted in important peak distortion. Therefore, 100% ACN was used as the dilution solvent in HILIC, resulting in satisfactory chromatographic results under optimized conditions, as shown in Figure 3.

### 2.3. Optimization of µ-SPE-PT Sample Preparation

A miniaturized SPE method development started with the preparation of home-made pipette tips filled with sorbents possessing different functionalities based on the previously described procedure [32]. Six different sorbents, including C_8_, C_18_, SDB, PGC, CX, and AX, were systematically tested first to individually evaluate retention of all target analytes on each sorbent type. Three layers of sorbent were used in these initial tests applying the generic protocol of activation with 100 μL ACN (3 min), conditioning by 100 μL water (5 min), and loading 100 μL solution of standards (15 min). The washing step was omitted at this stage of optimization to prevent the loss of analytes due to unoptimized conditions. Elution was accomplished with 100 μL of ACN (10 min). All these steps were carried out at 6 °C and a high centrifugation speed of 6500 RPM. Two fractions, i.e., loaded solution percolated through µ-SPE and eluate, were analyzed using the developed RP-UHPLC-MS/MS method to evaluate the effectivity of retention in individual sorbents. Each extraction experiment was run in triplicate. Most of the analytes were well retained on both C_8_ and C_18_ sorbents except for tenofovir and tenofovir monoester. Reduced recovery was achieved for saquinavir (55%), while almost no recovery was observed for maraviroc (0%). These reductions were attributed to the unoptimized elution step during this screening procedure. On the other hand, SDB and PGC sorbents were unsuitable for these multianalyte methods. They enabled strong retention and no elution was achieved even when using pure ACN. This especially applies to PGC. AX sorbent allowed efficient extraction for problematic maraviroc already when using the generic protocol. It worked well also for many other analytes except for didanosine, glecaprevir, tenofovir, tenofovir monoester, and zidovudine. On the other hand, CX sorbent did not enable efficient extraction in generic screening, although it has previously been reported to extract tenofovir and its analogues and thus required further in-depth optimization [33,34]. Therefore, two µ-SPE-PT protocols were optimized independently using C_18_ and combined with one of the ion exchange sorbents CX or AX.

In protocol 1, a combination of AX and C_18_ was used, placed in the tip following this order. The effect of the number of layers in the combination was evaluated based on the recovery of individual analytes in standard solution. Slightly lower overall recovery was obtained when using a combination of 1 × AX and 1 × C_18_ for some analytes including didanosine and zidovudine. Surprisingly, there was almost no effect for other analytes. Thus, the recovery was comparable with those of 2 × AX/C_18_ and 3 × AX/C_18_ tips for a large spectrum of analytes. The optimization was continued with 3 × AX/C18 sorbent in agreement with the previously published data [32] and to ensure sufficient quantity of the sorbent when using more complex matrix. It was necessary to use 90% ACN in the elution step because lower concentration decreased recovery for ledipasvir while 100% ACN compromised elution of abacavir, didanosine, and maraviroc. Tenofovir was not eluted at all from the AX sorbent upon these conditions. Acidification of elution solvent with 25 mmol/L formic acid finally allowed elution of tenofovir. Several agents, including water, water with different ammonia concentrations in the range of 1–10 mmol/L, 1–5% aqueous MeOH, and 1% aqueous ACN were tested in the washing step. Critical analytes remained the same, including didanosine, tenofovir, and zidovudine. These did not tolerate use of organic solvent in the washing step due to a significant decrease in their recovery. Most of the washing solvents reduced recovery of tenofovir. Thus, its extraction would only be possible without any washing step, which was inacceptable regarding to the composition of the biological medium.

**Figure 4:**
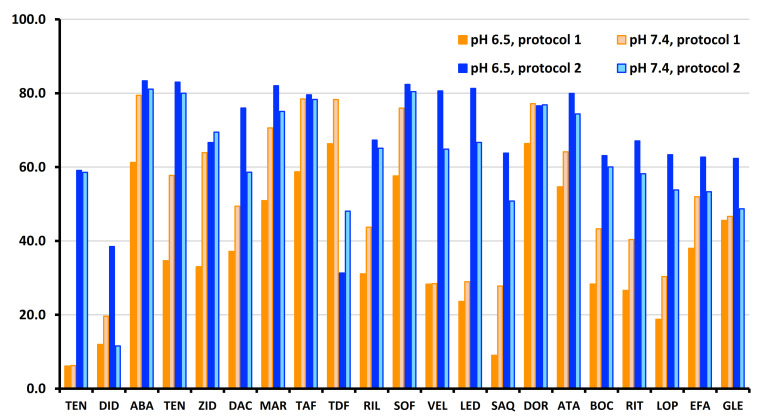
Comparison of two developed µ-SPE-PT sample preparation approaches based on AX/C_18_ and CX/C_18_ combinations of the sorbent and for two HBSS media of pH 6.5 and 7.4.

The transfer of the method optimized with standards to HBSS medium led to an important decrease in recovery to less than 50% for most of the analytes of our tested set and also revealed important difference in recovery between the two pH of the media. The recovery of tenofovir remained below 10%. Obviously, a reduction in recovery can be accepted in case of multianalyte method provided internal standard (IS) and quantitative approach setting ensure repeatable results and adequate sensitivity. Due to very high sensitivity of our method the recovery of 20–50% is not necessarily an issue provided it is repeatable. Therefore, protocol 1 was applicable for 19 target analytes but did not enable efficient extraction of tenofovir and didanosine as shown in Figure 4.

However, tenofovir is an important antiviral agent often included in combination antiviral therapies. Therefore, the second approach, protocol 2, was optimized with emphasis on efficient extraction of tenofovir and its derivatives as well as with the aim to improve the recovery of other analytes using sorbent combining CX and C_18_. Optimization steps similar to protocol 1 were repeated. The two-step elution was critical to solve the issue of simultaneous extraction of tenofovir and its derivatives simultaneously with other antivirals. Indeed, 80% aqueous MeOH with 1% ammonia was needed for the elution of tenofovir. In contrast, this elution solvent was not sufficient to accomplish elution of tenofovir disoproxil and other non-polar antivirals. Therefore, the second step had to be elution with neat ACN. To further improve retention of tenofovir on CX sorbent, acidification of sample with 1% formic acid was necessary prior to the loading step. The same agent was also found the most suitable washing solvent. This result agrees with the extraction principles when using CX sorbent. Simultaneously, these adjustments did not have any significant negative effect on the behavior of other tested analytes, as shown in Figure 4. The recovery from HBSS media obtained while using protocol 2 was improved for tenofovir, but also for other antivirals such as ledipasvir, velpatasvir, rilpivirine, saquinavir, boceprevir, ritonavir, and lopinavir that exhibited very low recovery in protocol 1. Moreover, the recovery was more consistent between the two HBSS media. Therefore, the protocol 2 was finally selected for method validation.

Due to the subsequent use of two different chromatographic methods, i.e., RP and HILIC, the dissolution and reconstitution in the mobile phase were the necessary final steps. The dissolution step was straightforward in HILIC, allowing the direct dissolution in 100% ACN. However, for RP analysis, the evaporated residue had to be reconstituted in the pure ACN part of the dissolution solvent first enabling dissolution of lipophilic antivirals and then completed with water to achieve the desired solution in 20% aqueous ACN.

### 2.4. Method Validation

Method validation was carried out in agreement with the requirements of ICH [35] and EMA [36] guidelines for bioanalytical method validation and with a description given in Section 3.6. The calibration range in RP-UHPLC-MS/MS was evaluated in the concentration range of 0.1–10,000 ng/mL to cover great differences in method sensitivity for individual analytes (Table 2). The calibration model was selected based on the error of back-calculated concentrations. The internal standard method and linear fit with logarithmic transformation was the most convenient calibration model. LLOQ of RP-UHPLC-MS/MS method ranged within 0.1–50 ng/mL. The LLOQ of 5–50 ng/mL obtained for didanosine, daclatasvir, glecaprevir, ledipasvir, and velpatasvir can be critical for some of the biological assays and resulted in insufficient method sensitivity. Matrix calibration curve was used to reduce the effect of matrix effects on quantitation. This approach allowed to achieve acceptable results of accuracy even in the presence of matrix effects. Indeed, despite using µ-SPE-PT clean-up, the matrix effects remained considerable for most of the analytes, see Section 2.5.

Concentration levels for determination of accuracy and precision were selected based on the determined method range and differed significantly for individual analytes. The results of method validation and individual concentration levels are shown in Table 2 for both HBSS media. Excellent method precision that met demanding criteria of the bioanalytical guidelines shows that the proposed µ-SPE-PT sample preparation method is a convenient approach for effective sample preparation even in multianalyte methods. Method precision criteria was not met only for two analytes, namely glecaprevir and ledipasvir. Method accuracy did not meet the criteria for five analytes, including daclatasvir, glecaprevir, ledipasvir, saquinavir, and velpatasvir at some of the validated concentration levels using one or both HBSS media. Thus, only 16 compounds can be considered as completely validated in RP-UHPLC mode. 

The evaluation of method selectivity has demonstrated that no significant response attributable to interfering components was observed at the retention times of our target analytes and their SIL-IS in the µ-SPE-PT extracted blank samples. Indeed, interference free chromatograms were obtained for 12 analytes. For nine analytes some responses were detected, but these were always lower than 20% of the analyte response at the LLOQ thus in compliance with the requirements of ICH guidelines.

The calibration range in HILIC-UHPLC-MS/MS method was evaluated in the range of 0.001–2000 ng/mL using matrix calibration curves (Table 3). Indeed, substantially improved sensitivity of about two orders of magnitude in HILIC was confirmed. Both linear and quadratic calibration curves with logarithmic transformation provided the best fit for HILIC calibration curves. The LLOQ in HILIC mode ranged from 0.001 to 10 ng/mL. The LLOQ of 10 ng/mL was obtained only for one compound, namely efavirenz. Besides this compound, all LLOQ are perfectly meeting the requirements of biological assays. Concentration levels were selected to meet ICH and EMA requirements. Moreover, more QC samples were prepared in HILIC to enable direct comparison of validation results at the same concentration level as in RP-UHPLC-MS/MS mode. These concentrations would be rather high related to the sensitivity and calibration range of the HILIC method. Nevertheless, the validation results were consistent through the whole validation range, confirmed the expectations, and allowed direct comparison with RP-UHPLC-MS/MS method. Table 3 shows the validation results and selected concentration levels in agreement with ICH and EMA requirements. The results are not shown for tenofovir and tenofovir monoester, since they were not soluble in neat ACN during the reconstitution step due to their high hydrophilicity. Except these two analytes, the validation results fully met the criteria of bioanalytical guidelines for both method accuracy and precision for all 19 analytes across the entire concentration levels. Similar to RP mode, important matrix effects were observed for most of analytes, but these were compensated by matrix calibration curve. Method selectivity was confirmed also in HILIC-UHPLC-MS/MS in the similar manner to RP method.

### 2.5. Comparison of the HILIC and RP Modes

RP and HILIC UHPLC-MS/MS methods were compared in terms of retention, separation selectivity, sensitivity, and method validation results. Both optimized methods enabled retention for all target compounds, while better separation selectivity was obtained in RP-UHPLC-MS/MS as shown in Figure 2 and Figure 3. This can be attributed to the narrower peaks obtained in RP mode corresponding to 0.03–0.04 min in all cases, except for boceprevir (0.08 min). In HILIC, the peak width was substantially larger, ranging from 0.03 to 0.07 min. Peak symmetry values obtained in RP ranged within 0.68–2.26 with only one value exceeding peak symmetry of 2. In HILIC, the symmetry factor ranged within 0.94–3.94 with five compounds exceeding the value of symmetry of 2. However, the repeatability of peak area never exceeded 10% RSD, thus reliable quantitative analysis was achieved for all compounds in both modes. On the other hand, substantially higher method sensitivity by a factor of 100× evaluated with matrix calibration curves was obtained with HILIC-UHPLC-MS/MS for most of the analytes. Even higher sensitivity was achieved for atazanavir (250×), glecaprevir (1000×), and ritonavir (500×). Sensitivity enhanced by a factor of 40–50× was obtained for doravirine, saquinavir, sofosbuvir, and zidovudine, while 20× higher sensitivity was observed for abacavir, boceprevir, didanosine, tenofovir alafenamide, tenofovir disoproxil, and velpatasvir. Only efavirenz has shown 5× lower sensitivity in HILIC-UHPLC-MS/MS compared to RP mode.

A comparison of validation results can be made based on Table 2 and Table 3. They clearly indicate that UHPLC-HILIC-MS/MS was more straightforward approach for the method validation allowing to achieve adequate results of accuracy and precision that meet rigorous criteria of bioanalytical guidelines EMA and ICH for 19 compounds, while 16 compounds provided these results in RP mode. Therefore, our HILIC method appears to be more convenient for multianalyte approach. However, it was not possible to accomplish the reconstitution step in HILIC for tenofovir and tenofovir monoester. Thus, RP mode remains the method of choice for these two compounds unless the evaporation and reconstitution step is omitted. The two methods have shown complementarity as the problematic analytes were not the same in both of them. Thus, one approach can replace the other in case of problems.

**Figure 5:**
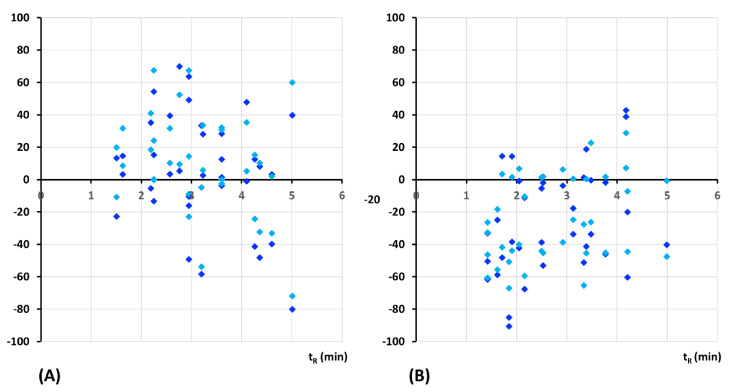
Matrix effects as a function of retention time. Comparison between RP-UHPLC-MS/MS method (**A**) and HILIC-UHPLC-MS/MS method (**B**) at two concentration levels, low QC (dark blue) and high QC (light blue) for HBSS media of pH 6.5 and 7.4.

A comparison of matrix effects in both methods is shown in Figure 5. Both methods suffered from important matrix effects that were expressed as signal enhancement in RP mode (Figure 5A) and signal suppression in HILIC mode (Figure 5B), respectively. In RP mode, the matrix effects of saquinavir and rilpivirine exceeded 100%. Thus, they are not displayed in Figure 5. In HILIC mode, matrix effects exceeding 100% were observed only for maraviroc. Despite important matrix effects, acceptable results of method accuracy were achieved due to matrix calibration curves used in data processing.

Moreover, our HILIC-UHPLC-MS/MS method is the first published HILIC approach used in multianalyte methods for analysis of antiviral drugs. In context of previously published methods for analysis of antivirals, our methods benefit from fast analysis, high number of antivirals analyzed simultaneously, high sensitivity, and more selective sample preparation approach. Indeed, to the best of our knowledge, no previously published report allowed simultaneous analysis of 20 or more antiviral drugs. These methods also used less selective methods of PP in sample preparation which was replaced with µ-SPE-PT in our study. Finally, both RP and HILIC mode allowed to achieve high sensitivity for all analytes with only few exceptions. Direct comparison of validation protocols of several recent works [23,24,25,26] shows that these works usually validated concentration levels of tens of ng/mL. Gouget et al. [24] have shown LLOQs in units of ng/mL, but they did not provide validation results. In our study, most of the analytes provided validation results meeting strict criteria of EMA and ICH at such low levels as 0.1–1 ng/mL in RP and even lower in HILIC.

## 3. Materials and Methods

### 3.1. Chemicals

The reference standards of antiviral drugs including abacavir (99.94%), atazanavir (99.95%), boceprevir (99.16), daclatasvir (99.84%), didanosine (99.81%), doravirine (99.79%), lopinavir (99.14%), rilpivirine (98.83%), ritonavir (100.0%), tenofovir alafenamide (99.75%), tenofovir disoproxil (99.16%), and zidovudine (99.91%) were obtained from TargetMol (Boston, MA, USA). Efavirenz (99.99%), ledipasvir (99.96%), maraviroc (99.95%), saquinavir (99.91%), sofosbuvir (99.97%), tenofovir (99.81%), and velpatasvir (99.95%) were purchased from MedChemExpress (Monmouth Junction, NJ, USA). Glecaprevir (98%) was obtained from AOBIOUS (Gloucester, MA, USA) and tenofovir monoester from Gilead Sciences (Foster City, CA, USA).

Stable isotopically labeled internal standards of abacavir-*d*_4_, atazanavir-*d*_6_, rilpivirine-*d*_6_, sofosbuvir-*d*_5_, maraviroc-*d*_6_, tenofovir disoproxil-*d*_6_, and tenofovir alafenamide-*d*_5_ were obtained from Toronto Research Chemicals (Toronto, ON, Canada). Tenofovir-*d*_6_ was obtained from Santa Cruz Biotechnology (Dallas, TX, USA). A list of analytes and their physicochemical properties is given in Table 1.

Acetonitrile (ACN) LC-MS grade and methanol, LC-MS grade were obtained from Merck (Prague, Czech Republic). Dimethylsulfoxide (DMSO) LC-MS grade was obtained from Sigma Aldrich (Prague, Czech Republic). Mobile phase additives, including acetic acid (100%, LC-MS grade), ammonium solution in methanol (4 mmol/L), and ammonium hydroxide (25% LC-MS grade), were purchased from Sigma Aldrich. Formic acid (≥99%, LC-MS grade) was obtained from VWR (Prague, Czech Republic). LC-MS grade water was prepared in the MilliQ RP device from Millipore (Burlington, MA, USA). InfinityLab Deactivator (medronic acid) was purchased from Agilent Technologies (Santa Clara, CA, USA). Individual components of the biological medium of Hanks balanced salt solution (HBSS), including albumin (≥95%), 4-(2-hydroxyethyl)-1-piperazineethanesulfonic acid (HEPES, 99%), sodium hydroxide (≥99%), and methanesulfonic acid (70%) were obtained from Merck and HBSS (10×) from Thermo Fisher Scientific (Prague, Czech Republic).

### 3.2. Instrumentation and UHPLC-MS/MS Analysis

All LC analyses, including both RP and HILIC separations, were carried out using identical UHPLC system ACQUITY UPLC I-Class PLUS (Waters, Milford, MA, USA) comprising a binary pump, an autosampler of fixed loop type with 10 µL sample loop, and a column thermostat. MS/MS detection was accomplished using triple quadrupole Xevo TQ-XS (Waters, Manchester, UK). This coupled system was controlled with MassLynx 4.1 software.

Screening a large set of conditions in both RP and HILIC was the first step in method optimization. Reversed-phase separations were carried out using generic gradient elution with water component and ACN as the mobile phase at a flow rate of 0.3 mL/min. The gradient started at 5% ACN and was linearly ramped to 98% ACN over 5 min followed by 2 min equilibration. The column temperature was 40 °C. Injection volume was 2 µL of the standard solution containing all 21 analytes dissolved in 20% ACN using a partial loop with needle overfill mode. Autosampler was washed using a weak wash liquid of 10% ACN and a strong wash liquid of neat ACN. Samples were stored in an autosampler at a temperature of 10 °C. Six different stationary phases, including BEH Shield RP18, BEH Phenyl, BEH C18, CSH fluorophenyl, CSH phenylhexyl, and CSH C18, all of them 2.1 mm ID, 100 mm length, and 1.7 µm particle size (Waters), were tested using the mobile phase containing 25 mmol/L formic acid as the aqueous component. Five different aqueous components, including formic acid, acetic acid, ammonia, ammonium formate pH 3, and ammonium acetate pH 9 at four different concentrations of 1, 5, 10, and 25 mmol/L were evaluated using the best stationary phase. Final optimized conditions of RP-UHPLC method were the following: BEH Shield RP18 stationary phase, original gradient elution using 25 mmol/L formic acid in water with the addition of 0.1% of medronic acid and ACN with addition 0.1% medronic acid, injection volume 2 µL, and flow rate 0.3 mL/min.

HILIC separations were carried out using HILIC generic gradient elution with water component and ACN at a flow rate of 0.3 mL/min. The gradient started at 98% of ACN and was linearly ramped to 50% ACN over 5 min followed by 3 min equilibration. The column temperature was 40 °C. Injection volume of the standard solution in ACN was 2 µL using a partial loop with needle overfill mode. Autosampler was washed using a weak wash liquid of 95% ACN and a strong wash liquid of 50% ACN. Samples were stored in an autosampler at a temperature of 10 °C. Ten stationary phases tested in HILIC mode involved BEH Amide (100 × 2.1 mm, 1.7 µm, Waters), BEH HILIC (100 × 2.1 mm, 1.7 µm, Waters), Cortecs HILIC (100 × 3.0 mm, 1.6 µm, Waters), Ascentis Express HILIC (100 × 2.1 mm, 2.7 µm, Sigma-Aldrich, Prague, Czech Republic), Ascentis Express OH5 (100 × 2.1 mm, 2.7 µm, Sigma-Aldrich,), Syncronis HILIC (100 × 2.1 mm, 1.7 µm, Thermo Fischer Scientific, Prague, Czech Republic), Kinetex HILIC (100 × 2.1 mm, 1.7 µm, Phenomenex, Aschaffenburg, Germany), Luna HILIC (100 × 2.1 mm, 3 µm, Phenomenex), Luna Omega SUGAR (100 × 2.1 mm, 3 µm, Phenomenex), and Zorbax HILIC plus (100 × 2.1 mm, 3.5 µm, Agilent Technologies, Santa Clara, CA, USA). Four water components, including formic acid, acetic acid, ammonium formate pH 3, and ammonium acetate pH 6 at two concentrations of 10 and 25 mmol/L, were examined with each stationary phase. The optimized HILIC method used Cortecs HILIC column and 25 mmol/L ammonium formate pH 3 (mobile phase A) and ACN (mobile phase B) in gradient elution with the described gradient program. Medronic acid (0.1 %) was added to both mobile phase components.

Triple quadrupole was operated in electrospray (ESI) positive and negative polarity switching mode. The ion source conditions were following: capillary voltage 1.0 kV in ESI^+^ and 1.5 kV in ESI^−^ in RP mode, and 2.0 kV in ESI^+^ and 2.5 kV in ESI^−^ in HILIC mode, ion source temperature 150 °C, nebulizer gas (nitrogen) 5.0 bar, desolvation gas (nitrogen) temperature 500 °C, desolvation gas flow 1000 L/h, and cone gas flow 150 L/h. SRM transitions were optimized individually for each analyte in ESI^+^ and ESI^−^ modes to select appropriate precursor ion, fragment ion, cone voltage, and collision energy. Cone voltages and ion source parameters were optimized in both HILIC and RP modes. Final SRM method settings are presented in Table 1.

### 3.3. Stock Solutions of Reference Standards

The stock solutions of individual antiviral drugs were prepared at a concentration of 1 mg/mL in agreement with the different solubility of compounds. The stock solutions of abacavir, daclatastvir, didanosine, tenofovir, tenofovir disoproxil, tenofovir monoester, and zidovudine were prepared in water. The stock solutions of atazanavir, glecaprevir, and sofosbuvir were prepared in 50% aqueous ACN. The stock solutions of rilpivirine was prepared in 90% aqueous ACN. The stock solutions of boceprevir, doravirine, efavirenz, ledipasvir, lopinavir, maraviroc, ritonavir, tenofovir alafenamide, and velpatasvir were prepared in neat ACN. The stock solution of saquinavir was prepared in dimethylsulfoxide. The stock solutions of the stable isotopically labeled internal standards were prepared in the same manner.

### 3.4. Biological Experiments to Evaluate Drug-Drug Interactions

A Caco-2 cell line-based assay was used as described previously to estimate the antivirals DDI at the intestinal barrier [37]. The Caco-2 human colon carcinoma cell line forms a polarized monolayer on filter insert (0.4-µm pore size, 12-mm diameter; Transwell 3401; Costar, Corning, NY, USA) mimicking the intestinal barrier. The monolayer separates two compartments, the apical (A, intestinal lumen) and basolateral (B, blood side). This setup allows studying drug permeability in both directions, i.e., from the intestine to blood (A to B) and from blood to the intestine (B to A). The Hanks’ balanced salt solution buffer (Thermo Fisher Scientific, Waltham, MA, USA) was used in both compartments. pH in the (A) compartment was adjusted to 6.5 using a methanesulfonic acid solution to mimic the acid microclimate of the small intestine, while pH of the buffer in the (B) compartment was 7.4 adjusted using HEPES to mimic the blood values. Evaluated drugs were added to the donor compartment (compartment A for A → B transport and compartment B for B → A transport) and samples were collected from the receiver compartment. To improve the reproducibility of the results, the receiver compartment always contained 1% bovine serum albumin as previously recommended [37]. Our experiment focused on the inhibitory effect of antivirals on the efflux transporter P-glycoprotein that is localized in the intestinal barrier and is a common site for the DDI. Rhodamine123 was used as a specific probe for this efflux transporter.

Hanks’ balanced salt solution (HBSS) used as the medium in biological experiments was prepared as follows: The basic buffer was prepared in 50 mL volumetric flask by the dissolution of 17.5 mg NaHCO_3_ in water followed by addition of 5 mL 10x concentrated HBSS and making up to volume. This basic buffer was subsequently titrated with HEPES or NaOH to achieve a pH of 7.4. An aliquot of 20 mL of the buffer pH 7.4 was mixed with 200 mg albumin when the buffer was used in the receiver compartment. The remaining solution was further titrated with methanesulfonic acid to achieve a pH of 6.5. An aliquot of 20 mL buffer pH 6.5 was mixed with 200 mg albumin in the case of the receiver compartment.

### 3.5. Sample Preparation

The μ-SPE-PT method for extracting antivirals from HBSS medium pH 7.4 and 6.5 was developed and optimized. Optimization of chemistry and quantity of the sorbent, as well as the composition of washing and elution solvents were carried out using one variable at a time approach. Sorbents Empore Octyl C8, Empore Octadecyl C18, Empore Styrene-Divinyl-Benzene (SDB-RPS), Empore Carbon-containing porous graphitic carbon (PGC), Empore Cation containing cation exchange (CX) group, and Empore Anion-SR containing anion exchange (AX) from 47 mm disks were cut, placed in pipette tips, and tested. All these sorbents were purchased from Merck. Centrifugation at 6500 rpm at a temperature of 6 °C was used to accomplish individual steps of extractions in pipette tips as described in our previous study [32]. Significant variability of physicochemical properties of the tested compounds required in depth optimization and some compromises. Therefore, two complementary μ-SPE-PT protocols were designed. The time needed to accomplish individual steps of the extraction process differed and are indicated for each step separately in brackets.

Protocol 1: A combination of three layers of AX and three layers of C_18_ was selected as the sorbent using the sorbent preparation procedure described in [32]. It was activated with 100 μL ACN (3 min), followed by conditioning by 100 μL water (5 min) and loading with 100 μL sample (7 min). The washing step was carried out with 100 μL 5 mmol/L ammonia in water (15 min). Elution was accomplished with 100 μL 25 mmol/L formic acid in 90% ACN (10 min). The eluate was evaporated to dryness using a vacuum evaporator (Concentrator Plus, Eppendorf, Prague, Czech Republic) in 15 min at 30 °C. The residue was reconstituted in 20% aqueous ACN for RP-UHPLC-MS/MS and in neat ACN for HILIC-UHPLC-MS/MS using an ultrasonic bath (Sonorex Digitec, Bandelin Electronic, Berlin, Germany).

Protocol 2: A combination of three layers of CX and three layers of C_18_ formed the most suitable sorbent using the sorbent preparation procedure described in [32]. It was activated with 100 μL MeOH (3 min), followed by conditioning by 100 μL water (5 min). The sample loading step involved mixing 100 μL sample and 100 μL 1% formic acid and loading of 200 μL of this pH-adjusted sample within 7 min. The washing step was carried out using 100 μL 1% formic acid (10 min). The two-step elution process was accomplished with (i) 100 μL 1% ammonia in 80% aqueous MeOH (3 min) and (ii) 100 μL ACN (20 min). The eluate was again evaporated to dryness using a vacuum evaporator for 35 min at 30 °C. The residue was reconstituted in 20% aqueous ACN for RP-UHPLC-MS/MS and in neat ACN for HILIC-UHPLC-MS/MS using an ultrasonic bath.

### 3.6. Method Validation

Both developed UHPLC-MS/MS methods were validated using µ-SPE-PT protocol 2 in the sample preparation step for the two Hanks balanced salt medium of pH 6.5 and pH 7.4. Parameters of linear range, accuracy, precision, matrix effects, the limit of detection (LOD), and the limit of quantitation (LOQ) were determined as requested by ICH [35] and EMA [36] guidelines for bioanalytical method validation. Biological samples of each matrix type were treated with µ-SPE-PT and subsequently analyzed using HILIC and RP UHPLC-MS/MS methods. The internal standard calibration with stable isotopically labeled internal standards was used for quantitation. Method selectivity was determined using blank matrices of HBSS media pH 6.5 and 7.4 treated by µ-SPE-PT procedure and measured by each RP-UHPLC-MS/MS and HILIC-UHPLC-MS/MS method. The results were evaluated following ICH [35] and EMA [36] recommendations.

Calibration range and calibration models were defined for each analyte individually in both HILIC and RP modes. The models were optimized so that the back-calculated concentrations of the calibration standards were always within ±15% of the nominal value at all calibration levels except for the lower limit of quantitation (LLOQ), where the back-calculated concentration could be ±20% [36]. Method accuracy and precision were calculated using quality (QC) control samples prepared at a minimum of four concentration levels including LLOQ, within three times LLOQ (low QC), within 30–50% of the calibration curve range (medium QC), and at least 75% of the upper limit of quantitation (ULOQ), which corresponded to high QC. All these analyses were carried out in five replicates with both matrices [35,36]. Due to substantially different physicochemical properties of target analytes and their ionization efficiency, these concentrations were very different among analytes and between the two tested HILIC and RP modes. Method accuracy must be within ±15% of error for all QC levels, except for LLOQ, where ±20% error is allowed. Method precision expressed as % RSD must be within 15% for all QC levels and within 20% for LLOQ, respectively. Matrix effects were determined using low and high QC concentration levels using post-extraction spiked matrix samples compared with the response of analytes in the standard solutions [35,36].

## 4. Conclusions

Development of new antiviral drugs, drug repurposing, optimization of antiviral therapy, and DDI evaluation require multianalyte analytical methods allowing to monitor a large spectrum of antivirals in biological matrices in a single analytical run with high sensitivity and selectivity. Two new UHPLC-MS/MS methods were developed using RP or HILIC mode in the simultaneous separation of a large spectrum of antiviral drugs. The main challenges in chromatographic method development were represented by analysis of hydrophilic tenofovir in both modes and by selection of appropriate dissolution solvent for the range of compounds with substantially different physicochemical properties. The compromises in analyte solubility and chromatographic peak shapes needed to be carefully addressed. The same applied for the optimization of µ-SPE-PT sample preparation approach, where the key feature was a combination of mixed-mode mechanism of C_18_ and ion-exchange sorbents of either AX or CX type to achieve effective extraction of a large spectrum of antivirals with different physicochemical properties. Especially C_18_ and CX combination has shown greater potential in multianalyte methods for antiviral drugs, which has not been reported yet.

In this paper, we show for the first time a comparison of applicability of RP and HILIC separation modes in analysis of antivirals. Despite higher separation selectivity achieved in RP mode compared to HILIC, matrix effects in the two methods were considerable. However, HILIC-UHPLC-MS/MS has shown several benefits in terms of substantially higher sensitivity, more straightforward method validation over wider dynamic range, and thus better applicability in this particular multianalyte method. Method sensitivity was incomparably higher related to the previously published reports dealing with LC-MS analysis of antivirals.

## Figures and Tables

**Table 1:** Physicochemical properties of antiviral drugs in this study and parameters of optimized SRM method.

Analyte	Abbreviation	Exact Mass (Da)	logP	pKa (Acidic)	pKa (Basic)	Acid-Base Properties	t_R_ in RP (min)	t_R_ in HILIC (min)	Precursor Ion Type	Precursor *m*/*z*	Fragment *m*/*z*	CV ^a^ in RP (V)	CV in HILIC (V)	CE ^b^ (eV)
abacavir	ABA	286.1542	0.39	15.43	5.80	basic	1.63	3.77	[M + H]^+^	287.2	191.0	15	15	20
atazanavir	ATA	704.3897	4.54	11.92	4.42	basic	3.60	2.05	[M + H]^+^	705.3	168.0	25	25	50
boceprevir	BOC	519.3421	1.70	12.44	−0.92	neutral	4.10	1.62	[M + H]^+^	520.3	308.1	35	35	25
daclatasvir	DAC	738.3853	5.11	12.47	5.40	basic	2.25	4.21	[M + H]^+^	739.2	565.1	10	10	40
didanosine	DID	236.0909	−0.35	10.94	2.76	neutral	1.51	3.34	[M − H]^−^	235.0	135.0	40	55	20
doravirine	DOR	425.0503	2.23	9.66	n/a	acidic	3.60	2.16	[M + H]^+^	426.0	314.9	40	55	20
efavirenz	EFA	315.0274	4.46	12.52	−1.49	neutral	4.60	1.42	[M + H]^+^	316.0	243.9	20	35	15
glecaprevir	GLE	838.2983	3.95	3.74	−1.20	acidic	5.01	1.42	[M + H]^+^	839.1	819.1	15	15	15
ledipasvir	LED	888.4134	6.71	11.22	5.32	basic	3.20	2.92	[M + 2H]^2+^	445.4	130.0	10	10	25
lopinavir	LOP	628.3625	4.69	13.39	−1.55	neutral	4.36	2.50	[M + H]^+^	629.3	155.0	15	15	40
maraviroc	MAR	513.3279	3.63	13.98	9.35	basic	2.25	4.99	[M + H]^+^	514.2	280.1	15	15	30
rilpivirine	RIL	366.1593	5.47	11.43	4.44	basic	2.95	1.71	[M − H]^−^	365.1	141.9	55	70	25
ritonavir	RIT	720.3128	5.22	13.68	2.84	neutral	4.26	2.53	[M + H]^+^	721.2	296.1	15	15	20
saquinavir	SAQ	670.3843	3.16	13.61	8.47	basic	2.95	4.18	[M + H]^+^	671.3	570.1	55	55	30
sofosbuvir	SOF	529.1625	1.28	9.70	n/a	acidic	3.23	1.91	[M + H]^+^	530.1	243.0	15	15	20
tenofovir	TEN	287.0783	−3.44	1.35	3.74	acidic	0.96	5.65	[M + H]^+^	288.1	176.1	15	15	25
tenofovir alafenamide	TNA	476.1937	1.88	11.36	3.74	basic	2.57	3.39	[M + H]^+^	477.1	270.0	15	15	30
tenofovir disoproxil	TDF	519.1730	2.65	n/a	3.74	basic	2.76	3.13	[M + H]^+^	520.1	270.0	15	15	25
tenofovir monoester	MONO	403.1257	−1.70	1.07	3.74	acidic	1.99	4.64	[M + H]^+^	404.1	270.0	15	45	20
velpatasvir	VEL	882.4065	5.11	11.71	5.36	basic	2.95	3.48	[M + 2H]^2+^	442.4	405.0	20	10	25
zidovudine	ZID	267.0968	−0.30	4.22	n/a	acidic	2.19	1.85	[M − H]^−^	266.0	223.0	30	35	10

^a^ CV—cone voltage; ^b^ CE—collision energy, Log P—partition coefficient. Blue lines show different ions than [M + H]^+^.

**Table 2:** Method validation results for RP-UHPLC-MS/MS.

		LOD (ng/mL)		LLOQ (ng/mL)		ULOQ (ng/mL)		r^2^ pH 7.4 pH 6.5		Concentration Levels (ng/mL) (*n* = 5)		Accuracy (%) pH 7.4 Buffer pH 6.5 Buffer		Precision (% RSD) pH 7.4 Buffer pH 6.5 Buffer		
					L1	L2	L3	L4	L1	L2	L3	L4	L1	L2	L3	L4
ABA	0.03	0.1	100	0.9991	0.1	0.2	20	50	16.3	4.7	−0.5	0.6	1.3	1.2	5.0	4.4
				0.9995					−1.4	6.9	7.9	3.1	2.7	5.3	4.9	2.1
ATA	0.15	0.5	100	0.9981	0.5	1	20	50	1.9	−9.1	1.7	7.1	2.7	3.0	2.2	3.8
	0.06	0.2	100	0.9988					12.4	13.3	14.8	11.8	2.0	1.0	3.3	2.4
BOC	0.6	2	1000	0.9996	2	5	200	500	6.7	4.3	−14.1	−14.7	4.7	3.1	1.8	2.3
	0.3	1	1000	0.9996	1	2			4.5	10.5	−3.3	−8.9	4.6	5.0	1.9	2.0
DAC	1.5	5	1000	0.9960	5	10	100	200	18.7	10.7	10.2	14.6	12.8	13.7	13.8	5.9
				0.9970			200	500	45.9	43.3	−27.6	−17.8	13.5	11.6	10.0	2.9
DID	6	20	1000	0.9990	20	50	100	500	5.8	−1.7	−4.0	−4.6	4.8	6.0	6.2	9.2
				0.9981					14.1	13.5	−1.7	−12.8	6.1	5.4	11.1	5.0
DOR	0.15	0.5	100	0.9994	0.5	1	20	50	−2.5	−7.8	−3.8	−6.4	6.1	6.4	2.5	3.2
	0.06	0.2	100	0.9992	0.2	0.5			3.1	1.5	13.5	7.3	8.5	2.8	2.9	1.6
EFA	0.6	2	1000	0.9989	2	5	10	50	10.0	−4.2	−4.2	−2.6	1.7	3.3	4.6	2.8
				0.9984					19.0	12.6	2.5	−10.2	6.4	9.6	8.9	10.6
GLE	3	10	1000	0.9949	10	50	200	500	−7.4	−17.0	−65.0	−63.4	25.0	25.6	9.9	12.2
	15	50	1000	0.9971	50	100			−52.7	94.7	90.5	68.8	26.2	5.7	1.9	4.6
LED	1.5	5	200	0.9991	5	10	100	200	−2.7	−14.8	0.2	1.6	7.5	6.1	8.3	12.6
				0.9980			50	100	−15.3	3.0	−4.3	185.5	13.4	16.1	20.0	16.6
LOP	0.3	1	1000	0.9998	1	2	10	50	9.5	0.7	−13.6	−14.9	8.6	6.6	6.1	2.9
				0.9997			200	500	13.5	8.1	13.3	11.3	5.9	5.9	4.5	5.1
MAR	0.3	1	100	0.9970	1	5	20	50	−4.2	−14.5	−4.2	−1.0	1.7	3.6	2.9	5.6
				0.9979					13.3	−6.8	5.3	3.9	3.5	2.9	3.6	3.4
RIL	0.6	2	1000	0.9986	10	50	100	200	3.6	−6.6	−4.6	−6.2	8.1	7.7	8.8	2.8
			500	0.9972			200	500	20.0	7.4	0.8	−9.1	2.8	1.6	7.1	5.9
RIT	0.3	1	1000	0.9996	1	2	100	200	11.2	5.8	−10.7	−11.6	9.7	6.0	4.5	2.6
				0.9996			200	500	9.6	9.4	13.0	9.8	6.5	4.9	4.3	3.4
SAQ	0.06	0.2	100	0.9982	0.2	0.5	20	50	26.5	35.1	31.9	54.3	6.0	14.5	6.5	5.4
				0.9985			1	5	−6.0	9.0	6.7	10.0	11.8	10.8	9.8	13.7
SOF	0.03	0.1	100	0.9993	0.1	0.2	20	50	10.4	5.6	1.2	2.3	6.4	2.9	2.0	3.3
	0.06	0.2		0.9994	0.2	0.5			19.4	9.3	6.2	1.4	2.1	4.5	3.7	2.6
TEN	0.3	1	100	0.9924	1	5	20	50	18.1	−13.0	−5.1	9.0	9.7	6.0	4.5	2.6
				0.9960					19.3	−9.3	1.8	3.8	5.6	4.0	5.8	4.4
TNA	0.03	0.1	100	0.9996	0.1	0.2	20	50	6.8	2.9	−5.4	−2.6	4.8	4.2	0.8	2.3
				0.9998					5.4	10.9	5.3	3.8	3.7	5.4	2.9	2.8
TDF	0.03	0.1	100	0.9993	0.1	0.2	20	50	16.3	3.3	−6.0	−4.3	9.6	9.8	3.5	3.8
				0.9997					10.7	15.0	6.4	2.7	12.1	7.1	3.2	3.7
MONO	0.03	0.1	100	0.9995	0.1	0.2	20	50	3.0	8.6	1.3	2.9	5.1	6.2	3.5	3.1
				0.9995					0.7	7.3	13.4	13.1	10.1	5.6	3.0	3.3
VEL	6	20	10000	0.9976	20	50	100	200	−13.4	−14.6	−27.6	−23.1	14.0	14.6	15.0	15.0
	3	10	1000	0.9975	10	20			−13.2	−12.6	−17.0	−13.6	17.3	10.7	6.3	9.4
ZID	0.3	1	1000	0.9986	1	2	200	500	−15.8	1.1	12.4	7.4	17.8	9.8	5.4	3.5
				0.9985					−14.7	13.9	14.3	13.1	11.2	6.5	3.8	6.9

**Table 3:** Method validation results for HILIC-UHPLC-MS/MS.

	LOD (ng/mL)	LLOQ (ng/mL)	ULOQ (ng/mL)	r^2^ pH 7.4 pH 6.5	Concentration Levels (ng/mL) (*n* = 5)				Accuracy (%) pH 7.4 Buffer pH 6.5 Buffer				Precision (% RSD) pH 7.4 Buffer pH 6.5 Buffer			
					L1	L2	L3	L4	L1	L2	L3	L4	L1	L2	L3	L4
ABA	0.0015	0.005	20	0.9999	0.005	0.01	5	10	8.0	4.0	0.5	−1.0	4.1	6.3	1.4	1.6
				0.9998					−4.0	−5.0	0.1	0.1	5.7	3.7	1.7	2.4
ATA	0.0006	0.002	20	0.9998	0.002	0.005	5	10	10.0	12.0	4.3	2.4	12.4	4.0	1.1	1.3
				0.9947					15.0	−4.0	7.6	9.1	17.4	5.7	1.5	1.5
BOC	0.015	0.05	200	0.9998	0.05	0.1	50	100	10.6	9.8	1.2	0.7	2.7	7.7	1.6	1.8
				0.9995					0.0	−4.4	5.9	−1.5	2.3	2.6	1.3	1.0
DAC	0.015	0.05	200	0.9997	0.05	0.1	50	100	2.4	7.9	0.3	0.7	2.9	3.9	2.9	0.6
				0.9989					1.2	2.9	2.2	4.3	4.9	3.8	1.9	2.0
DID	0.3	1	2000	0.9991	1	2	500	1000	5.6	−0.2	3.1	1.7	4.3	8.9	9.1	2.5
				0.9993					9.8	−3.3	−0.5	−10.8	10.0	6.2	2.6	3.8
DOR	0.0015	0.005	20	0.9998	0.005	0.01	5	10	2.0	1.0	−0.3	−7.5	8.2	7.3	1.0	1.0
				0.9995					−2.0	−1.0	0.1	−8.2	4.6	9.0	4.8	2.9
EFA	3	10	2000	0.9995	10	50	500	1000	7.4	12.5	5.2	0.1	4.6	5.0	2.9	1.1
				0.9991					7.3	14.8	14.6	5.6	7.1	7.5	4.1	3.6
GLE	0.015	0.05	200	0.9994	0.05	0.1	50	100	9.0	10.5	−11.7	5.4	3.4	4.6	5.1	1.4
				0.9994					−4.4	13.4	9.0	10.8	9.3	10.5	7.7	3.9
LED	0.015	0.05	200	0.9994	0.05	0.1	50	100	8.0	10.8	7.0	2.2	8.2	4.0	3.0	0.8
				0.9995					−4.6	−9.2	7.3	5.6	4.2	5.3	5.3	1.4
LOP	0.003	0.01	20	0.9996	0.01	0.02	5	10	7.0	−2.5	7.1	9.8	4.2	6.3	2.3	2.4
				0.9998					3.0	7.0	−1.4	0.5	5.5	2.0	4.6	1.7
MAR	0.0015	0.005	20	0.9975	0.005	0.01	5	10	−1.7	5.0	8.1	7.4	9.3	4.8	1.7	0.6
				0.9982					8.0	2.0	6.7	1.4	4.1	4.4	1.1	1.9
RIL	0.006	0.02	20	0.9974	0.02	0.05	50	100	15.0	0.0	4.0	9.4	11.9	7.1	2.1	1.4
				0.9968					10.0	4.0	2.6	−2.0	20.3	12.9	1.3	2.9
RIT	0.0006	0.002	20	0.9949	0.002	0.005	5	10	5.0	2.0	7.4	11.0	10.6	4.4	2.7	2.3
				0.9980					0.1	−2.0	0.9	1.3	0.1	4.6	4.2	1.4
SAQ	0.0015	0.005	20	0.9994	0.005	0.01	5	10	−2.0	10.0	−8.0	−0.8	4.6	10.2	5.8	0.9
				0.9995					−8.0	−3.0	4.1	11.1	14.2	12.9	3.4	3.7
SOF	0.0015	0.005	20	0.9981	0.005	0.01	5	10	6.0	5.0	6.3	4.6	5.2	4.8	0.4	1.7
				0.9966					6.0	−2.0	2.8	1.8	8.4	2.8	1.0	1.0
TNA	0.0015	0.005	20	0.9997	0.005	0.01	5	10	8.0	9.0	2.7	0.6	4.1	2.1	1.3	1.4
				0.9994					4.0	−3.0	1.1	2.8	5.3	2.8	2.2	1.1
TDF	0.0015	0.005	20	0.9998	0.005	0.01	5	10	2.0	−3.0	3.7	0.3	4.4	2.8	1.9	1.7
				0.9997					−6.0	−3.0	−2.6	−1.3	5.8	4.6	0.9	3.0
VEL	0.15	0.5	500	0.9976	0.5	1	100	500	5.4	6.1	14.3	7.2	2.2	2.7	1.7	2.3
				0.9967					−2.2	−8.5	14.2	4.5	7.5	4.0	1.6	3.5
ZID	0.006	0.02	100	0.9994	0.02	0.05	50	100	13.7	2.7	0.9	−11.9	13.2	4.1	7.3	2.5
				0.9995					13.0	4.3	−8.5	−5.6	15.3	7.1	4.9	7.5

## Sample Availability

Samples of the compounds are not available from the authors.
